# Selective Production of Xylooligosaccharides by Xylan Hydrolysis Using a Novel Recyclable and Separable Furoic Acid

**DOI:** 10.3389/fbioe.2021.660266

**Published:** 2021-04-09

**Authors:** Jianglin Zhao, Xiaotong Zhang, Xin Zhou, Yong Xu

**Affiliations:** ^1^Jiangsu Co-Innovation Center of Efficient Processing and Utilization of Forest Resources, College of Chemical Engineering, Nanjing Forestry University, Nanjing, China; ^2^Key Laboratory of Forest Genetics and Biotechnology, Ministry of Education, Nanjing Forestry University, Nanjing, China; ^3^Jiangsu Province Key Laboratory of Green Biomass-Based Fuels and Chemicals, Nanjing, China

**Keywords:** xylooligosaccharides, xylan, furoic acid, hydrolysis, bio-oxidation, electrodialysis

## Abstract

Xylooligosaccharides (XOS) have gained considerable attention worldwide as prebiotics due to their immune-strengthening activity and beneficial gut bacteria development and can be produced from xylan-rich resources by acid hydrolysis. The present study proved the organic acid hydrolysis to be beneficial for XOS yield. In this study, a recyclable and separable organic acid, i.e., furoic acid, was used for hydrolyzing xylan to produce XOS, and the response surface methodology design was applied to maximize the XOS yield; the results indicated that the quadratic model terms of the interaction between reaction temperature and hydrolysis time showed the most significant impact on XOS yields (*P* < 0.05). The predicted maximum yield of XOS was 49.0% with 1.2% furoic acid at 167°C for 33 min, being close to the experimental value (49.2%), indicating that the fitted models were in good agreement with the experimental results. Meanwhile, the primary byproducts, including xylose and furfural, were concurrently bio-oxidized into xylonic acid and furoic acid by *Gluconobacter oxydans* and separated by electrodialysis. Subsequently, the furoic acid with low solubility (<3.7%, 25°C) was recovered by natural crystallization. The above results indicate that the use of multi-steps contributes to sustainable XOS production by furoic acid.

## Introduction

The emerging health consciousness has concurrently increased the awareness and pivoted the food preferences of consumers to use natural, healthier, and sustainable food resources, such as prebiotics and preventive medicines (Aachary and Prapulla, 2011; Santibáñez et al., 2021). The common prebiotics, defined as a non-digestible food ingredient, consists of oligosaccharides and polysaccharides that are resistant to human digestive enzymes hydrolysis but beneficial to the host by selectively limiting the gut microbiota growth (Gao et al., 2020). Xylooligosaccharides (XOS), the sugar-oligomer prebiotics, composed of β-1,4-linked xylose units, are regarded as potential food ingredients due to their multidimensional physiological properties on human health and livestock, such as lowering cholesterol and risk of colon cancer and improving calcium absorption and intestinal canal function (Samanta et al., 2015). Thus, the XOS market price varies from the US $25/kg to $50/kg; and efficient industrial-based technologies with high XOS production are developing rapidly to fulfill market needs (Jayapal et al., 2013; Mhetras et al., 2019).

Currently, the common technologies for XOS production from xylan hydrolysis are categorized as the acid hydrolysis, the enzymatic hydrolysis, or combination of these two approaches (Carvalho et al., 2013; Hong et al., 2019); and abundant supply of renewable and low-cost lignocellulosic materials guarantee stable production of xylan (Zhao et al., 2020). The potential high market value has driven researchers to develop advanced methods to convert xylan to XOS. The enzymatic hydrolysis requires a relatively prolonged time and unique conditions for enzyme storage, and XOS output is lower (Samanta et al., 2012; Jnawali et al., 2017). In contrast, acid hydrolysis is a practical and conventional industrial technology (Lin et al., 2017). With the action of acid, xylan can be effectively degraded into oligosaccharide and monosaccharide, which can be harvested in the liquid that separated after the acid hydrolysis. Thus, various mineral acids and organic acids were employed to hydrolyze xylan. However, mineral acids (strong acids) as catalyst will lead to the accelerated reaction of xylose into furfural due to the higher activation energy for the dehydration; in addition, the mineral acid application will cause a larger inorganic waste stream and mostly gypsum (Kootstra et al., 2009). Correspondingly, the organic acids exhibit the desirable characteristics, including selective hydrolysis with more oligomeric sugars and lower degradation byproducts (Bian et al., 2014; Cesaro et al., 2020; Guo J. et al., 2020).

Nowadays, various organic acids, such as formic acid, acetic acid, maleic acid, gluconic acid, and oxalic acid, have been introduced to acid-hydrolyze xylan or xylan-rich lignocellulosic materials for XOS production (Guo X. et al., 2020; Han et al., 2020). Lin et al. (2017) developed an oxalic acid hydrolysis strategy for producing XOS using beechwood xylan as feedstock and achieved a yield of 39.31%. Zhang et al. (2017) employed acetic acid to produce XOS with a yield of 45.9%. In addition, Zhou et al. (2019a) found that gluconic acid also performed good ability to decompose sugarcane bagasse-xylan into XOS with a yield of 53.2%. Besides, organic acid byproduct stream could be easily applied in animal feed, fertilizing soil, and co-firing installations (Lin et al., 2017).

Furoic acid (FA) is a heterocyclic carboxylic acid comprising a five-membered aromatic ring and a carboxylic acid group; the dissociation constants of oxalic acid are 3.16 (pKa at 25°C); thus, FA also can release H^+^ at high temperature to depolymerize xylan into the oligosaccharide and monosaccharide. FA has been widely used in food products as preservative and flavoring agent; the solubility of FA was only 37 g/L in 25°C. Dai et al. (2021) reported that FA was an effective catalyst to pretreat the sugarcane bagasse into XOS with a yield of 45.6% and also proved that FA could be recovered easily by cooling and natural crystallization from recovered acid solution. Although FA can result in a high XOS yield using sugarcane bagasse as material, the XOS content was only 11.9 g/L, and the pre-hydrolyzate from acid hydrolysis of sugarcane bagasse contains various byproducts, such as formic acid, acetic acid, phenolic compounds, pectin, and crude proteins (Zheng et al., 2021); it makes direct production of XOS from biomass not feasible. Thus, in this context, and considering the target of high concentration and purity XOS production, the present work studied its production condition that depends on FA-assisted hydrolysis of alkaline soluble xylan from corncob.

## Materials and Methods

### Preparation of Xylan

Corncob as feedstock was collected from Dongtai, Jiangsu, China. The dried corncob mainly comprises 34.1% glucan, 30.4% xylan, 18.7% lignin, and 4.2% ash content. The xylan was prepared from the milled corncob in triplicate using 7% sodium hydroxide and then subjected to steam treatment for 45 min at 120°C with a solid to liquid ratio of 1:7. Then the alkali solubilized xylan was first filtered using a zero-filter paper followed by Whatman filter paper 40 and precipitated using 95% ice-cold ethanol (Samanta et al., 2012). The precipitate was further dried in a forced hot air oven at 60°C until constant weight. The xylan was analyzed according to the National Renewable Energy Laboratory’s standard method (Sluiter et al., 2008), and the purity and recovery rate of the extracted xylan were 71.6 ± 1.9% and 82.1 ± 1.6%, respectively.

### Response Surface Method Design and Optimization

Design-Expert (Version 11.0), a response surface analysis software, was selected to design the experiment; and an appropriate point was selected according to the experimental results (Neto et al., 2020). There were three independent variables in the experiment: reaction temperature (130–170°C), acid concentration (1–3% w/w), and hydrolysis time (10–60 min). The values of these independent variables are listed in Table 1. The experiments were run 15 times in triplicate according to the Box–Behnken design matrix. The combination test design principles used three factors: reaction temperature (***X*_1_**), retention time (***X*_2_**), and FA concentration (***X*_3_**).

**TABLE 1:** Different combinations of independent variables of software design and experimental results.

Variables			Responses
Reaction temperature (°C)	FA concentration (% w/w)	Hydrolysis time (min)	XOS yields (%)
130	1	10	2.50
130	1	60	15.6
130	2	35	10.4
130	3	60	37.6
130	3	35	18.6
150	3	10	11.3
150	3	35	44.3
150	2	60	37.7
150	2	35	38.9
150	1	60	43.1
170	2	10	34.5
170	1	35	48.0
170	3	35	14.4
170	2	35	31.2
170	1	60	24.9

*XOS, xylooligosaccharides; FA, furoic acid.*

In a 30-ml steel reactor tube made of 316 stainless, 1.0 g of xylan solids was firstly added, and then 10 ml of 1–3% FA solution was mixed in the tube and stirred uniformly. Subsequently, sealed reactor tube was immersed in oil baths at preset temperatures for varying durations based on the experimental design. Once the hydrolysis reaction was completed, the reactor tube was immediately taken out and cooled to room temperature. The supernatant was collected by centrifuging the mixture at 10,000 × *g* for 5 min and then subjected for XOS analysis. All experiments were carried out in triplicate. The total XOS content, which contains DP 2–6 oligomers, was calculated as a response variable. Regression analysis of the experimental data and the response surface plots were calculated by using statistical software Design-Expert (Version 11.0). One-way analysis of variance (ANOVA) and Duncan’s multiple range test (*P* < 0.05) were carried out to analyze the statistical significance. The relationship between the response variable and independent variables was calculated through the following quadratic polynomial equation.

$$Y=a_{0}+\sum a_{i}\,X_{i}+\sum a_{\mathrm{ii}}\,X_{i}^{2}+\sum a_{\mathrm{ij}}\,X_{i}\,X_{j}$$

where, **Y** is the yield of XOS (%), ***a*_0_** represents a constant term, ***X*** (***X*_*i*_** and ***X*_*j*_**) represents independent variables, and ***a*_*i*_**, ***a*_*ii*_**, and ***a*_*ij*_** represent coefficients of linear, quadratic, and interaction parameters, respectively.

The coefficients in the second-order polynomial were calculated based on the experimentally obtained data, and then the predicted optimal conditions for the maximum yield of XOS were obtained. Lastly, an additional experiment with three replications was conducted to verify the validity of the predicted optimum values by the program.

### Xylose and Furfural Bioconversion

*Gluconobacter oxydans* 621H, purchased from the American Type Culture Collection (ATCC), was employed to bio-catalyze xylose and furfural into xylonic acid (XA) and FA. Strain was maintained on sorbitol-agar slant (5.0 g/L of yeast extract, 20 g/L of agar, and 10 g/L of glycerol) at 4°C. *G. oxydans* inoculum was cultivated in 100 g/L sorbitol and 10 g/L yeast extract media in a 1-L Erlenmeyer flask and cultured for 24 h at 220 rpm and 30°C. The cell pellets of *G. oxydans* were then harvested by centrifugation (6,000 × *g*, 5 min, 4°C). The xylan-hydrolyzate was filtered by a 0.45-μm filter Millipore for sterilization. In a 250-ml Erlenmeyer flask (50 ml of xylan-hydrolyzate, 220 rpm, and 30°C) was loaded 2 g/L *G. oxydans* cells for bio-oxidizing xylose and furfural, and samples were taken at suitable intervals for component analysis (Zhou et al., 2019b).

### Furoic Acid Recovery

The bipolar membrane electrodialysis crystallization method was employed for FA recovery. The instrument consisted of four chambers: acid, salt, alkali, and electrode chamber. To increase the electrical conductivity throughout the process, 0.3 mol/L of sodium sulfate was added to the electrode chamber. After the mixed solution of XOS and FA entered the salt chamber, the FA was collected in the acid chamber through an electric current. However, XOS was still trapped in the salt chamber, and the corresponding alkali compounds were formed in the alkali chamber. Finally, XA, FA, and XOS were separated from the mixed solution by this method.

### Analytical Methods

Xylose, xylobiose (X2), xylotriose (X3), xylotetraose (X4), xylopentaose (X5), and xylohexaose (X6) in liquid were simultaneously determined by high-performance anion exchange chromatography (HPAEC) (Dionex ICS-3000, United States) coupled with a CarboPac^TM^ PA200 column (Xu et al., 2013). Furfural and FA were analyzed by a high-performance liquid chromatograph (HPLC) (Agilent 1200, United States) coupled with an Aminex Bio-Rad HPX-87H column (Bio-Rad Laboratories, United States). The yield (%) of furfural, xylose, and XOS was calculated as follows: the content of furfural, xylose yield, and XOS in hydrolyzate is divided by the initial xylan content and multiplied by 100.

## Results and Discussion

### Xylooligosaccharides Production by Furoic Acid-Assisted Hydrolysis

**FIGURE 1:**
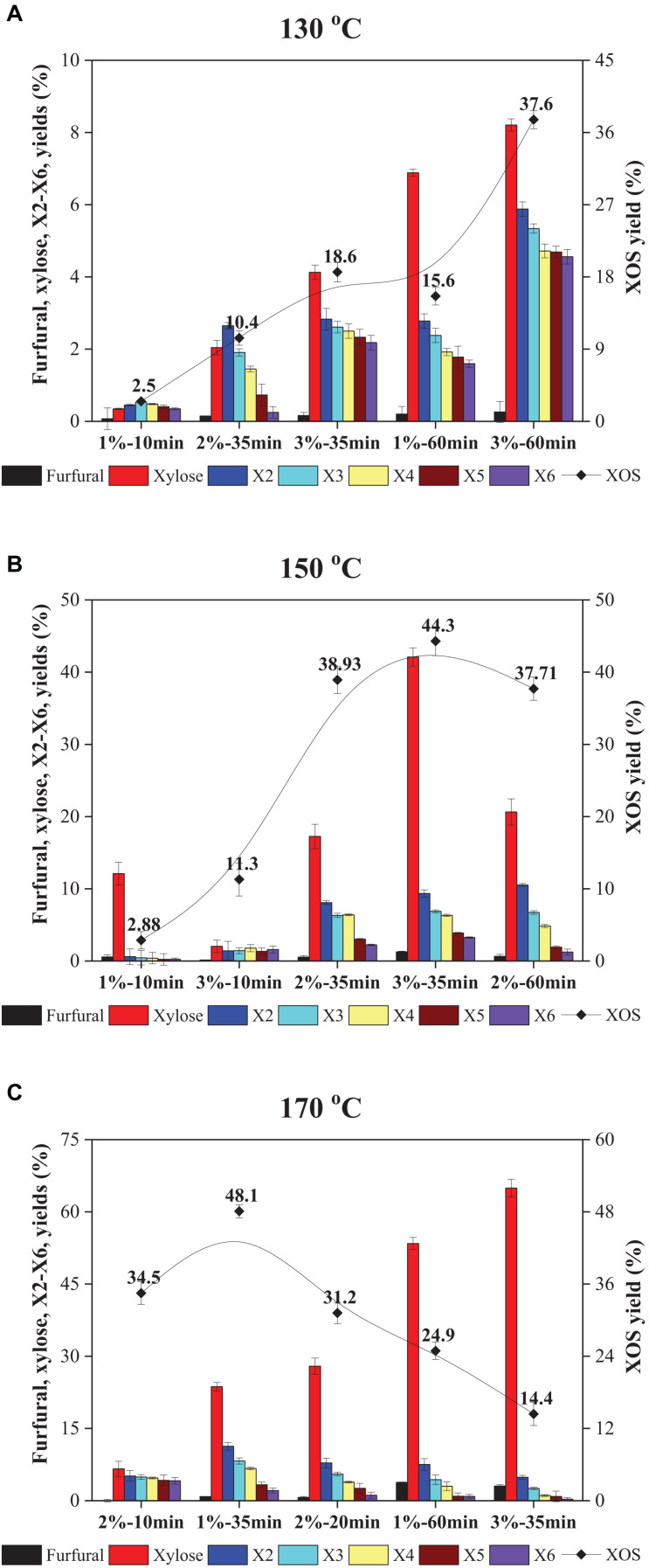
Yields of furfural, xylose, X2–X6, and XOS in FA hydrolyzate of xylan at different FA concentrations, times, and temperatures: **(A)** 130°C; **(B)** 150°C; and **(C)** 170°C. XOS, xylooligosaccharides; FA, furoic acid.

Generally, the degradation rate of xylan is positively correlated with the reaction temperature, acid concentration, and hydrolyzed time. The total amount of XOS liberated from xylan during the hydrolysis is a major parameter to determine the effectiveness of these factors. In the present study, the highest acid concentration was 3% because the solubility of FA was below 4%. Previous studies have shown that the hydrolysis of xylan was difficult to be controlled while the acid-assisted hydrolysis temperature was over 170°C, because xylan was easy to be acid hydrolyzed at the case of high temperature, and excessive temperature results in higher activation energy for the dehydration; that is, is easy to accelerate the dehydration of xylose into furfural; therefore, excessive temperature usually leads to large amounts of byproducts (xylose and furfural) being generated (Akpinar et al., 2009; Guo et al., 2019). When hydrolysis temperature was lower than 130°C, xylan is particularly hard to be degraded; lower temperature will lead to a long reaction time, even more than 60 min (Han et al., 2020). According to the preliminary experiment, the acid concentrations of 1–3% and reaction time of 10–60 min at 130–170°C were selected for xylan hydrolysis. In this work, the response surface methodology (RSM) was employed in this work to maximize the XOS yield, and the coefficients of a mathematical model were determined. An experimental design with a total of 15 different runs and the corresponding responses are given in Table 1. After the acid hydrolysis, xylose and furfural were determined to compare the effects of different hydrolysis conditions, and the yields are shown in Figures 1A–C.

As depicted in Figure 1A, the XOS yield at relatively low FA concentration, temperature, and time was low. The XOS produced by 1% FA after 10 min at 130°C was only 2.5%. That tardy enhancement in XOS yield by extension of hydrolysis time or enhancement of FA concentration at 130°C can also be seen. When the reaction was conducted at 130°C, the highest yield of XOS was only 37.6%, which should use 3% FA for 60 min, while the XOS yield showed a quick increase from 18.6% (130°C, 3% FA, 35 min) to 44.3% (150°C, 3% FA, 35 min) by improving the reaction temperature, which is depicted in Figures 1A,B. A similar observation was also obtained by temperature improvement of 150°C to 170°C; the XOS shows a rapid increase from 11.3% (150°C, 3% FA, 10 min) to 34.5% (170°C, 2% FA, 10 min), as displayed in Figures 1B,C.

The above results suggested that low temperatures decelerated the xylan hydrolysis, whereas higher temperatures significantly accelerated the degradation of xylan into XOS. This phenomenon was also shown by Lin et al. (2017). Therefore, the temperature range of 150–170°C was considered as the optimum temperature for XOS formation. Additionally, it was observed that XOS yields obtained similar results with 2% FA at 150°C for 35 min (38.9%) and 60 min (37.7%). Prolonged retention time resulted in further degradation of X5 and X6, which means that higher amounts of X2 and X3 would occur. In the case of 1% FA as a catalyst at 170°C, it showed a significant decrease in XOS yields from 48.1% (35 min) to 24.9% (60 min) and a significant increase in xylose yield from 18.1% to 41.4%. Therefore, it was inferred that high reaction temperature and prolonged hydrolysis times would decline the XOS content due to further degradation of XOS to xylose and furfural under these optimum reaction conditions. Generally, the highest XOS yield from organic acid hydrolysis is in the range of 35–50% (Lin et al., 2017; Zhang et al., 2017; Zhou et al., 2019a). Thus, all results indicated that FA was comparable with the reported organic acids (acetic acid, oxalic acid, maleic acid, citric acid, and gluconic acid) and was a practical assist-catalyst.

### Fitting Models

All results indicated that the distribution of xylose and X2–X6 was significantly dependent on FA concentration, reaction temperature, and hydrolysis time. Moreover, the XOS decreased with the increasing acid concentration and/or prolonging hydrolysis time at high temperature; meanwhile, it reduced the XOS purity by forming xylose and furfural in the xylan-hydrolyzate. It is worth mentioning that the hydrolysis of xylan took prolonged time in the lower reaction temperature, suggesting the process to be economically unfavorable and that high reaction temperature with short hydrolysis time is much more preferable for oligosaccharide formation with desired DP and lower byproduct yields.

In this study, Design-Expert 11.0 software was employed to establish relationships between the independent and dependent variables. The fit summary report was generated by Design-Expert, which recommended that the quadratic models were suitable for the XOS yields from the experimental responses, which followed the regression equations.

$$Y=-1500.59+18.86\,X_{1}+20.59\,X_{2}+4.50\,X_{3}-0.47\,X_{1}\,X_{2}-0.029\,X_{1}\,X_{3}+0.47\,X_{2}\,X_{3}-0.054\,X_{1}^{2}+6.11\,X_{2}^{2}-0.013\,X_{3}^{2}$$

**FIGURE 2:**
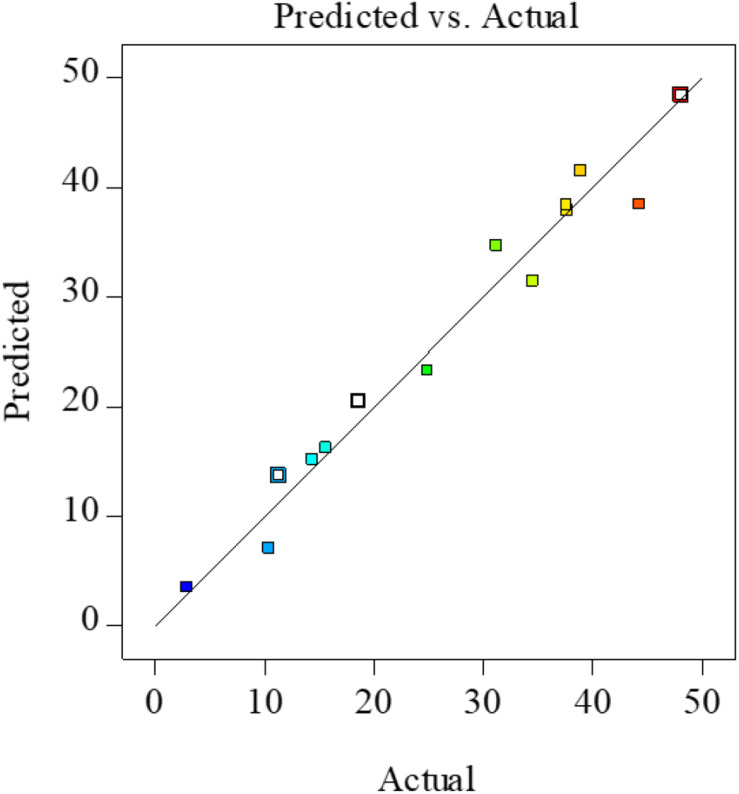
Actual vs. predicted XOS yields from FA hydrolysis of xylan. XOS, xylooligosaccharides; FA, furoic acid.

It is well known that the determination coefficient *R*^2^ is defined as the ratio of the explained variation to the total variation and demonstrates the agreement between the observed and predicted results, which used to estimate goodness of fit of the regression equation, and it should be over 0.80 (Nath and Chattopadhyay, 2007). In the present study, *R*^2^ was 0.928, indicating a close relationship between the predicted theoretical values and the experimental results; this similarity could also be verified by the high correlations between the observed and predicted values in Figure 2.

Analysis of variance of the model showed the *F*-value and *P*-value to be 20.96 and 0.0019, respectively. The *F*-value of 20.96 and the *P*-value of less than 0.050 suggest the model to be significant. In this case, the analysis of XOS yields showed the *P*-values of ***X*_1_**, ***X*_3_**, ***X*_1_*X*_2_**, ***X*_1_*X*_3_**, ***X*_1_**^2^, and ***X*_3_**^2^ to be 0.0021, 0.0031, 0.0024, 0.0020, 0.0038, and 0.0064, respectively, indicating their significant effects on XOS yields. Besides, the *P*-values of ***X*_1_**, ***X*_2_**, and ***X*_3_**, were 0.0013, 0.0025, and 0.0598, respectively. *P*-value is used to check the order of significance for independent variables affecting the XOS yield, where smaller *P*-values mean higher significance (Xue et al., 2016). Thus, according to the *P*-values, the effect of independent variables on XOS yields in order to sort is as follows: Reaction temperature, hydrolysis time, and FA concentration.

**FIGURE 3:**
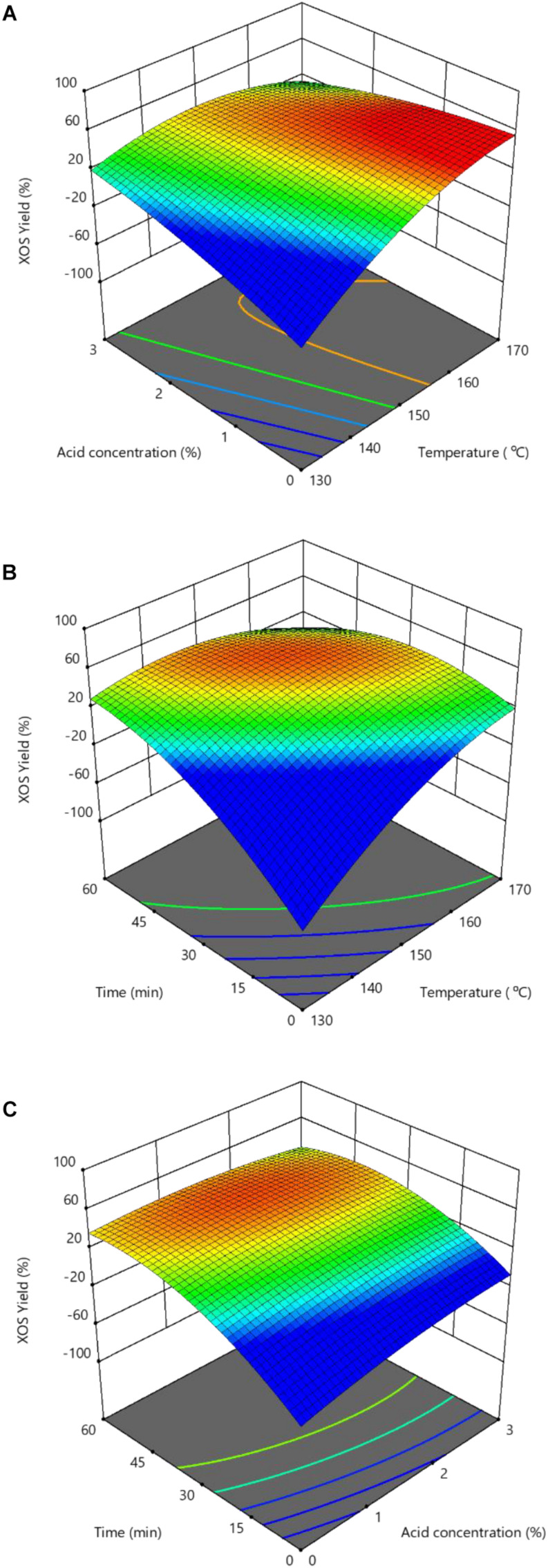
Response surface showing the effects of independent variables on XOS yields: **(A)** Reaction temperature and hydrolysis time; **(B)** Reaction temperature and FA concentration; and **(C)** Hydrolysis time and FA concentration. XOS, xylooligosaccharides; FA, furoic acid.

Two-dimensional contour plots and three-dimensional response surface plots were generated from Design-Expert software and depicted in Figures 3A–C. These results demonstrated that the reaction temperature effect was more significant on XOS yield than the hydrolysis time and acid concentration. Figures 3A–C also illustrate the optimal conditions, indicated by the red area. This resulted in the highest XOS yield with a predicted value of approximately 49.0%, which could be achieved at a higher temperature of 167°C with 1.2% FA for 33 min. The experimentally obtained real yield of furfural, xylose, and XOS under the predicted optimized conditions was 1.71 ± 0.09%, 32.3 ± 2.6%, and 49.2 ± 3.5%, respectively. Further, the real contents of X2–X6 were achieved at 12.10 ± 0.81, 10.31 ± 0.64, 6.58 ± 0.42, 4.01 ± 0.36, and 2.10 ± 0.28 g/L, respectively; and total XOS was 35.1 ± 2.5 g/L. Apparently, this predicted the XOS yield to be similar to the optimal experimental XOS yield, suggesting the accuracy of the established model to predict the XOS yield.

The mass balance of the two-step process showed that 40% xylan in corncob (contains 30.4% xylan) could be converted into value-added XOS; that is, approximately 125 g XOS could be harvest from 1,000 g of corncob material. The totality of the present results indicated that XOS preparation from corncob with FA-assisted hydrolysis was a profitable option.

### Furoic Acid Recovery by a Combination of Bio-Oxidation and Electrodialysis

Xylooligosaccharides, as higher value-added products, therefore, are our prior target. The above results showed that acid hydrolysis at a relatively high temperature could effectively depolymerize xylan into XOS, which suggested that FA was an effective catalyst for XOS production. However, a great deal of xylose and a small quantity of furfural, as main byproducts, were simultaneously generated during the FA hydrolysis process. Under the optimum conditions, 49 g/(100 g xylan) XOS was produced; as well as 32 g/(100 g xylan) xylose and 2 g/(100 g xylan) furfural were accumulated in the xylan-hydrolyzate. In addition, 12 g/L of FA as catalyst was also preserved in the xylan-hydrolyzate. Generally, commercial XOS products comprise DP 2–6 oligomers with 70–95% purity (Moure et al., 2006; Singh et al., 2018). Thus, both catalyst (FA) and byproducts (xylose and furfural) should be removed to improve purity of XOS. Previously, Zhou et al. (2017) have reported that xylose and furfural could be bio-oxidized into XA and FA by *G. oxydans* with 100% yield. In addition, Cao et al., successfully separated and collected XA from fermented broth by using electrodialysis (Cao and Xu, 2019). These studies suggested that the combination of bioconversion and electrodialysis could effectively remove xylose and recover FA.

**FIGURE 4:**
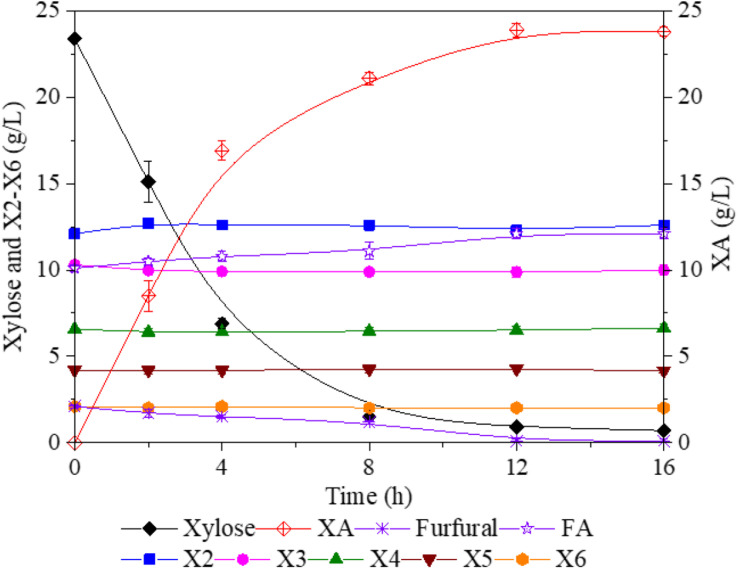
Bioconversion of xylose and furfural into XA and FA by *Gluconobacter oxydans*. XA, xylonic acid; FA, furoic acid.

After FA hydrolysis, the pH of xylan-hydrolyzate was below 2.5, which would significantly suppress the activity of *G. oxydans*. Thus, the xylan-hydrolyzate was prior adjusted to pH 6.5 with NaOH and then subjected to *G. oxydans* for biotransform xylose and furfural into XA and FA, respectively. During the fermentation, NaOH was also used to maintain pH, which resulted in XA⋅Na and FA⋅Na formed in the hydrolyzate. After 12 h of fermentation, 24 g/L of XA with a yield of 97.1% was accumulated in the broth. Besides, 14.1 g/L of FA retained in the solution; of these, 2.1 g/L of FA was from the furfural conversion. These results indicated that xylose and furfural were exclusively and effectively bio-oxidized into XA and FA without further catabolism. Meanwhile, the X2–X6 curves, shown in Figure 4, confirmed that XOS could not be utilized by *G. oxydans*, allowing the XOS, FA⋅Na, and XA⋅Na to be retained for the downstream separation.

**FIGURE 5:**
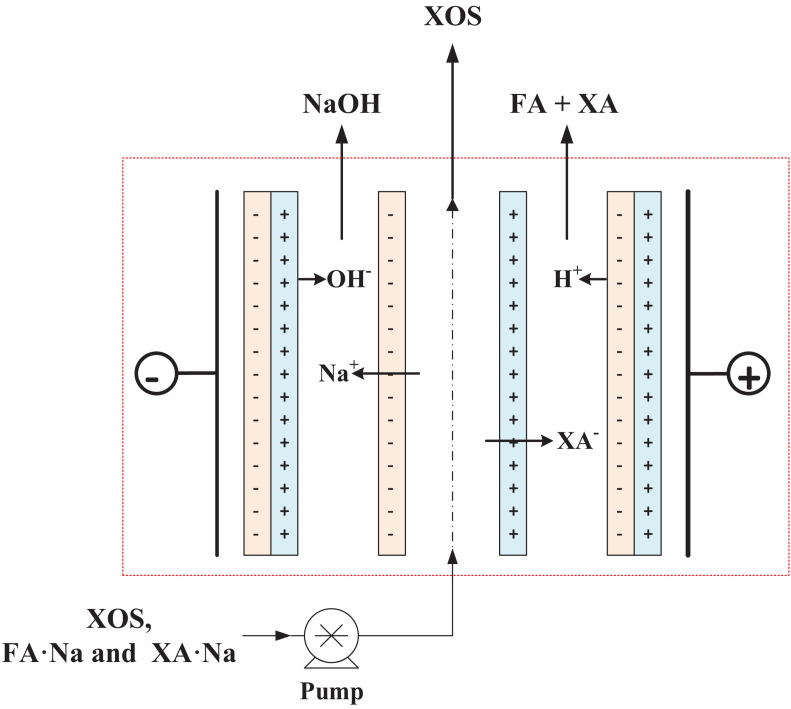
Schematic of bipolar membrane electrodialysis for FA and XA separation. XA, xylonic acid; FA, furoic acid.

Subsequently, the pH of hydrolyzate after bio-oxidation by *G. oxydans* was first adjusted to 6.5–7.0 for enhancing electrodialysis efficiency; and the mixture of the FA⋅Na, XA⋅Na, and XOS was then subjected into salt chamber of electrodialysis for XOS, FA, and NAOH recovery. The schematic of bipolar membrane electrodialysis for FA and XA separation is shown in Figure 5. The electrodialysis process was conducted at 50 mA/cm^2^ of current density; and the pH of hydrolyzate in salt chamber was maintained at 6.0–7.0 in the process of electrodialysis (Zhou and Xu, 2019). After approximately 90 min of electrodialysis, 95.1% of FA and 97.6% of XA were recycled, and 98% XOS were retained in the salt chamber solution. Overall, electrodialysis with bipolar membranes is one of the most promising and convenient methods to isolate the ionic salts and recycle sugar compounds (Xu and Huang, 2008; Pan et al., 2017). The solubility of FA was only 37 g/L (25°C) due to the increased acid concentration in the acid chamber during the electrodialysis process. Thus, FA could be recovered easily by natural crystallization in the acid chamber. The recovery of FA not only improved the XOS purity but also reduced the waste stream and the output cost, which can potentially replace the organic acid-assisted XOS production.

## Conclusion

In the present study, a recyclable and separable FA was employed to hydrolyze xylan into XOS. The effects of temperature, FA concentration, and hydrolysis time were assessed by RSM design, defining the maximum XOS production point. Finally, 35.1 g/L of XOS at the yield of 49.2% was produced with 1.2% FA at 167°C for 33 min. In conclusion, this study verified the feasibility of XOS production from FA, new organic acid hydrolysis combining recycled techniques. This model offers a practical framework for economical use of resources.

## Data Availability Statement

The original contributions presented in the study are included in the article/Supplementary Material, further inquiries can be directed to the corresponding author/s.

## Author Contributions

JZ: methodology, data curation, and formal analysis. XZa: investigation, data curation, and resources. XZo: conceptualization, supervision, validation, and writing—review and editing. YX: project administration and funding acquisition. All authors contributed to the article and approved the submitted version.

## Conflict of Interest

The authors declare that the research was conducted in the absence of any commercial or financial relationships that could be construed as a potential conflict of interest.
