# Supplementary material for: Selective Production of Xylooligosaccharides by Xylan Hydrolysis Using a Novel Recyclable and Separable Furoic Acid
Source: Front Bioeng Biotechnol. 2021 Apr 9;9:660266. doi: 10.3389/fbioe.2021.660266 (PMC8062972; doi:10.3389/fbioe.2021.660266)

Supplementary Material

**Supplementary Figure 1.** Graphic Abstract for schematic diagram of xylooligosaccharides production from xylan hydrolysis using furoic acid.


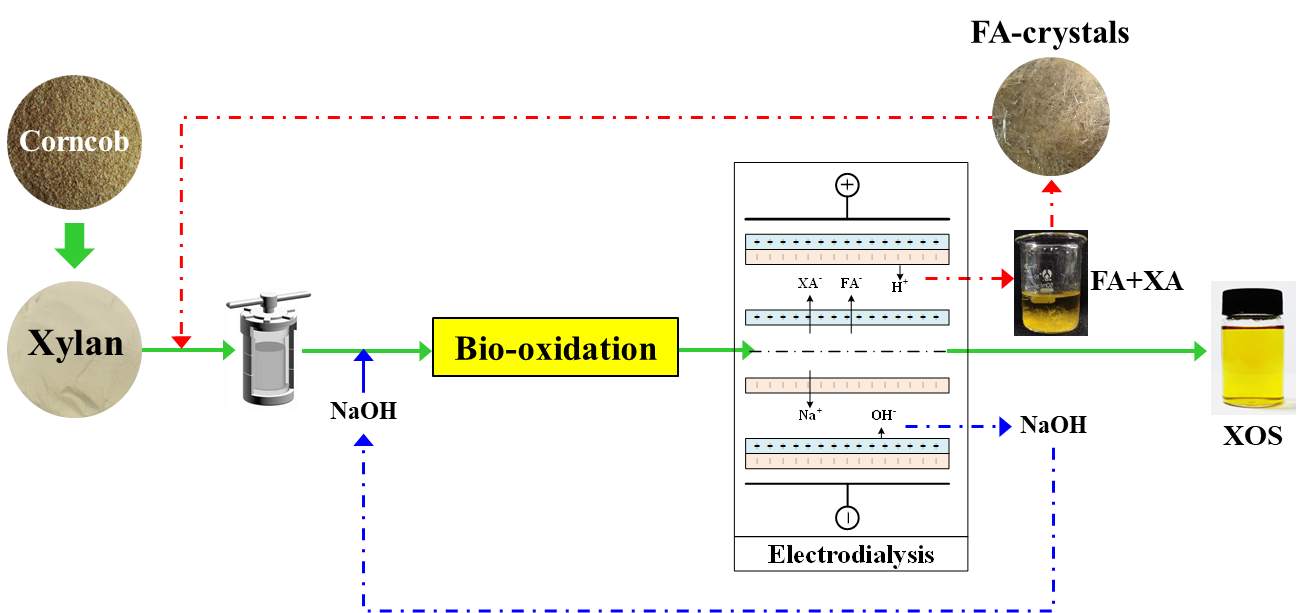


**Figure 2.** Chromatogram of high-performance anion exchange chromatography (HPAEC) for XOS sample from FA-assisted acidic hydrolysis

**Figure 3.** The photograph of actual electrodialysis equipment.


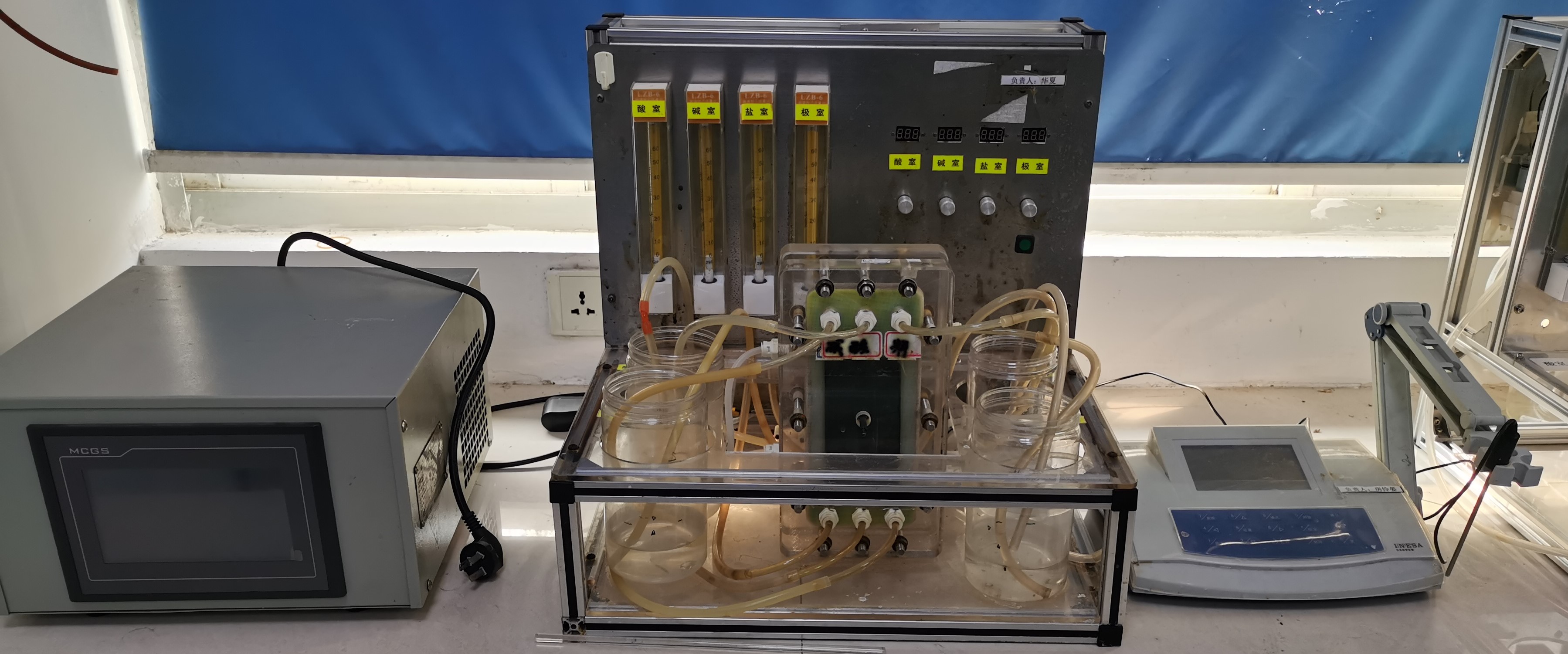

Supplement: Supplementary file 1 [file Data_Sheet_1.docx]
